# Two Moral Arguments for a Global Social Cost of Carbon

**DOI:** 10.1080/21550085.2018.1448038

**Published:** 2018-03-13

**Authors:** Kian Mintz-Woo

**Affiliations:** a Philosophy, University of Graz, Graz, Austria

Donald Trump’s executive order on energy limits the costs and benefits of carbon to domestic sources. The argument for this executive order is that carbon policies should not be singled out from other policies as globally inclusive. Two independent arguments are offered for adopting a global social cost of carbon. The first is based on reinforcing norms in the face of commons tragedies. The second is based on the limitations of consequentialist analyses. We can distinguish consequences for which probabilistic indifference is appropriate. The mechanisms for global effects for carbon are well-understood, whereas most policy effects are primarily domestic.

In March 2017, the Trump administration issued an executive order (EO) on Promoting Energy Independence and Economic Growth (White House, 2017). Among other things, the EO limits the included costs and benefits of carbon to domestic sources. More specifically, this applies to the social cost of carbon (SCC) or the monetized cost to society of increasing contemporary emissions by a marginal ton of carbon dioxide.^1^ In practice, when determining the estimated damages from that marginal ton of carbon dioxide, this change holds that only the expected domestic impacts are to be included.

While the choice between domestic or global SCCs may appear arcane or unimportant, here are two reasons to think otherwise. First, the actual impact of this rule change is significant. Since greenhouse gases are long-lived and disperse freely in the atmosphere, the effects of that marginal ton fall on many different countries in a highly varied manner; restricting the effects to any single country ignores international impacts, resulting to a lower SCC. Second, it acts as synecdoche for the broader orientation of the Trump administration. The question that the administration poses is whether nationals should care about foreigners.

The morally interesting puzzle is why, as the Obama administration and others contended, *carbon* regulatory analysis should appeal to (at least some) global costs and benefits, but not analyses of other targets of regulation (Fraas et al., 2016; Gayer & Viscusi, 2016). Other potential targets include, but are not limited to, terrorism, illegal drugs and infectious diseases (Gayer & Viscusi, 2017). This is on its face surprising; what could be so special about the SCC when typical regulatory impact analyses only consider domestic impacts?

I offer two responses to this moral puzzle. The first is based on reinforcing norms in the face of commons tragedies with observable responses. The relevant fact about carbon is that its negative *effects* are *predominantly* global and not local. The second is based on the fundamental limits of consequentialist analysis. The relevant fact for this argument is that the physical mechanisms by which international impacts are propagated are *well understood*. If we can justify a global SCC from different moral premises, it is a morally robust claim.

First, climate change is a global commons problem (e.g. Revesz et al., 2017; Vanderheiden, 2008) or one with global negative externalities (e.g. Stern, 2015), meaning that costs of an action to others are not born by the actor.^2^ Global commons problems of this sort threaten to generate tragedies of the commons (Hardin, 1968): there are strong incentives against cooperating (i.e. acting in such a way where most benefits flow to others) and in many versions of such problems it is rational to defect (i.e. not cooperate). However, climate change is different from the standard version in several key aspects; two important ones are that it is iterated and that states can see what policies others are adopting.

This last point is key: with respect to climate change, states can *observe* whether other states are co-operating by instituting global SCCs. The states which have instituted SCCs have in almost all cases introduced ones with values that are greater than if they were purely domestic (cf. Howard & Schwartz, 2017, Appendix B). In the light of this key point, we can endorse the following principle:

*Reinforcing norms*. When facing commons problems where the value of cooperating accrues disproportionately to others *and* the problem is iterated *and* other members of the commons are known to be cooperating, in order to generate and reinforce cooperative norms, one also ought to cooperate.

The duty *Reinforcing norms* gives rise to is defeasible and may fail to apply when there are *extremely* weighty contrary moral considerations. However, since tragedies of the commons are so damaging and this principle can be used to avoid these tragedies, I suggest it has considerable normative force. Note that *Reinforcing Norms* does not give rise to the duty to be the *first* co-operator in a commons problem, but it does give rise to moral duties to *follow* other co-operators, even if one does not expect this to be the best strategic response. My claim is thus weaker than claims in the literature that countries (such as the United States) morally must *lead* with respect to climate change in light of their abilities and historical emissions (Maltais, 2014; Shue, 2011).

One could object that this should apply similarly with respect to many other problems like terrorism and infectious diseases. If one country fails to contain the spread of a zoonosis or a terrorist organization, that failure can impose further significant costs on the global community. However, emissions impose much larger global than domestic costs. Sometimes, terrorism or infectious diseases spread globally and, sometimes, they impose large global costs, but, unlike damages from emissions, the effects from these bads can be—and in the vast majority of cases are—limited geographically. For comparison, consider regulations requiring vaccinations. Like climate change, when people are properly vaccinated, many of the benefits in terms of herd immunity do not directly accrue to those vaccinated (Wang, Clymer, Davis-Hayes, & Buttenheim, 2014) and, like climate change, vaccination rates can be influenced by regulation (Orenstein & Hinman, 1999). However, the impact of reduction of vaccination predominantly applies regionally (Wang et al., 2014). In this manner, these other problems are not, or not as clearly, global commons problems, since the benefits of regulation are primarily domestic.

Second, we can consider an argument for a global SCC from fundamental limitations in consequentialism. Cost–benefit analyses assume consequentialism, meaning that the decision to act in a particular way depends only on the consequences of that act—here, the act of regulating carbon dioxide emissions. One could be worried that consequentialists cannot completely evaluate *any* act because the actual consequences of those actions can be distant in time and place and very great either in positive or negative ways (Lenman, 2000). The usual response is to appeal only to the consequences which are expected or believed to result from the act. What about the other consequences? If we lack understanding of mechanisms by which we could understand how acting in a particular way or refraining from acting in the same way would lead to a particular outcome, then we do not need to consider those effects in our analysis. This is because consideration of those effects would not reverse judgements made only by considering expected effects; intuitively, their value would *cancel out* between acting or refraining (Greaves, 2016).

This position also justifies distinguishing between acts like regulating carbon and regulating to prevent other bads like terrorism or illegal drugs. The international effects of regulation aimed at these other bads are much more speculative than the international effects of decreased carbon emissions. This is because of the well-understood basic physical mechanisms of climate change (IPCC, 2014, SPM 1.3). Since we understand the basic physical mechanisms well, we cannot justify excluding them from our cost–benefit analyses; therefore, we ought to include all (i.e. the global) effects into our SCC. In contrast, the mechanisms by which terrorism and illegal drugs spread globally are complex and contingent; to the extent our epistemic condition with respect to these bads make it unclear whether given regulation helps prevent or spread them *internationally*, the foregoing claims mean that we are justified in not considering the global effects with respect to these regulations.

These two arguments justify adoption of a global SCC. They show that, at least in this respect, contra the claims of the Trump administration, we should consider the global implications of our actions.

## Disclosure statement

No potential conflict of interest was reported by the author.

## Funding

This work was funded by the Austrian Science Fund (FWF) under research grant W1256-G15 (Doctoral Programme Climate Change–Uncertainties, Thresholds and Coping Strategies).

## Notes

1. While there are small benefits associated with smaller levels of increased carbon dioxide on items like crop yield, the majority of effects would be costs (IPCC, 2014, SPM 1.3). Therefore, for simplicity, I refer to costs, but this caveat applies throughout.

2. For our purposes, we do not need to distinguish these cases; the argument applies regardless of which way it is framed. For ease of exposition, I use the commons framing throughout.
